# Clinical Efficacy and Safety Comparison of Rivaroxaban and Dabigatran for Nonvalvular Atrial Fibrillation Patients Undergoing Percutaneous Left Atrial Appendage Closure Operation

**DOI:** 10.3389/fphar.2021.614762

**Published:** 2021-06-18

**Authors:** Xiaoye Li, Xiaochun Zhang, Qinchun Jin, Ying Xue, Wenjing Lu, Junbo Ge, Daxin Zhou, Qianzhou Lv

**Affiliations:** ^1^ Department of Pharmacy, Zhongshan Hospital, Fudan University, Shanghai, China; ^2^ Department of Cardiology, Zhongshan Hospital, Fudan University, Shanghai, China

**Keywords:** novel oral anticoagulation, percutaneous left atrial appendage closure, device-related thrombosis, transesophageal echocardiographic imaging, clinical efficacy and safety

## Abstract

**Objective:** Due to the clinical complexity of warfarin, novel oral anticoagulation (NOAC) has been a feasible and safe alternative anticoagulant approach during left atrial appendage closure (LAAC). This study was designed to compare the efficacy and safety of rivaroxaban and dabigatran for nonvalvular atrial fibrillation patients undergoing percutaneous LAAC.

**Methods:** One single and prospective cohort study was performed among patients who received anticoagulation with dabigatran or rivaroxaban. All patients were medicated with a 3-month course of NOAC to facilitate device endothelialization, followed by dual antiplatelet therapy until 6 months, then lifelong aspirin after discharge. Repeated transesophageal echocardiography was scheduled to evaluate thrombosis formation on occluders and thrombus dissolution ability.

**Results:** A total of 262 consecutive patients were initially enrolled. A final number of 250 patients were analyzed; two patients were excluded due to procedure failure and 10 patients had a loss of follow-up; 97 were from the dabigatran group and 153 from the rivaroxaban group. Three patients (1.9%) in the rivaroxaban group and eight (8.2%) in the dabigatran group were experiencing device-related thrombosis (DRT) events during follow-ups. Cumulative Kaplan–Meier estimates showed that the incidence of DRT was lower under rivaroxaban medication during the 6-month follow-ups (*p* = 0.038*, OR = 3.843, 95%CI: 0.991–14.836). The transesophageal echocardiography (TEE) results showed that the average length and width of DRT in the rivaroxaban group was significantly lower compared with that in the dabigatran group (2.16 vs. 1.60 mm, *p* = 0.017*, and 1.71 vs. 1.30 mm, *p* = 0.003*, respectively). The thrombosis dissolved after the switch from dabigatran or rivaroxaban to warfarin within the target range, represented by the average length and width of thrombus with the cooperation of secondary TEE for the dabigatran and rivaroxaban groups (0.64 vs. 0.40 mm, *p* = 0.206, and 0.43 vs. 0.27 mm, *p* = 0.082, respectively). No significant difference was found between the two groups with respect to the levels of coagulation parameters, cardiac function, and bleeding events.

**Conclusion:** Compared to dabigatran, post-procedural rivaroxaban anticoagulation might be advantageous in preventing DRT complications expected after LAAC, without increasing the risk of hemorrhage.

## Introduction

Currently, percutaneous left atrial appendage closure (LAAC) has become an effective and safe surgical method for the prevention of stroke, mainly in patients diagnosed with nonvalvular atrial fibrillation (NVAF) and who could not adhere to long-term anticoagulant therapy (Reddy et al., 2013; Iskandar et al., 2016; Reddy, 2018). It has been reported that thrombosis in the left atrium can significantly increase stroke risk, and almost 90% of the identified left atrial thrombosis is located in the left atrial appendage (LAA) (Saw et al., 2019). The LAAC operation proposed a concept mainly attributed to the combination of the reduction in thromboembolic events and potential benefits concerning bleeding events when oral anticoagulation (OAC) is stopped after occluder implantation (Boersma et al., 2017; Reddy et al., 2017).

Similar to other implants, there is a necessary period for complete endothelialization on implanted occluders after exposure to circulating blood. After occluder implantation, local response to tissue injury occurred, leading to formation of thrombus, which is observed as a similar situation in devices with endothelialization process. Therefore, active postoperative antithrombotic therapy is required to prevent device-related thrombosis (DRT) (Dukkipati et al., 2018; Fauchier et al., 2018; Asmarats et al., 2019). Due to the clinical complexity of warfarin, which has been suggested as a standard anticoagulant approach during LAAC, strong interest was developed in alternative therapies with novel oral anticoagulants (NOACs) in the peri- and post-procedural settings after occluder implantation (Enomoto et al., 2017). The current guidelines recommend anticoagulation with NOAC for patients ineligible for warfarin to facilitate device endothelialization followed by dual antiplatelet therapy until 6 months, then lifelong aspirin (Glikson et al., 2020). However, there was a significant inter-individual variability on anticoagulation before complete endothelialization on the device, which might add to the uncertainty of the duration of antithrombotic therapy during this vulnerable time for DRT.

Recently, clinical trials have prompted a warning against advising anticoagulation with dabigatran in patients with mechanical heart valve replacement mainly due to the enhancement of thrombin receptor expression on platelets and consecutively increased platelet reactivity (Eikelboom et al., 2013). Meanwhile, rivaroxaban, a selective Xa inhibitor, has been confirmed to decrease the clot formation induced by thrombin, resulting in the reduction in thrombin burst during the propagation phase of the coagulation cascade, and remained favorable for patients with vascular thrombosis in the COMPASS trial (Sharma et al., 2019). Currently, some cases reported DRT formation during anticoagulation with dabigatran in patients undergoing percutaneous LAAC (Li et al., 2020). Therefore, we hypothesized that the use of rivaroxaban or dabigatran might influence thrombosis formation. Thus, the objective of our study was to analyze the clinical outcomes of a different regimen of antithrombotic therapy among Chinese patients after LAA occluder implantation.

## Methods

### Study Design and Population

A single-center, prospective cohort study was performed among NVAF patients who underwent percutaneous LAAC operation between January 2017 and December 2018. This study was approved by the Medical Ethics Committee of Zhongshan Hospital. The diagnostic criteria of NVAF were consistent with the European Society of Cardiology (ESC) guidelines (Kirchhof et al., 2016). To be enrolled in this study, patients who had a higher risk of stroke, who had transient ischemic attack, or had a systemic embolism risk score (CHA_2_DS_2_-VASc ≥ 2) were not suitable for long-term anticoagulation. All enrolled patients were eligible to undergo LAAC operation with occluders and had never undergone atrial fibrillation ablation before. These patients were medicated with either dabigatran or rivaroxaban before the implantation procedure to reduce stroke/systemic embolism risk. The exclusion criteria of this study mainly included the following: (1) a bleeding history and comorbidity with hemorrhagic disease, (2) severe hepatic and renal dysfunction, and (3) discontinuation of anticoagulation with dabigatran and rivaroxaban. Based on drug administration upon admission, patients were categorized into two groups: the dabigatran group (110 mg b.i.d., the only available dosage) or the rivaroxaban (15 mg q.d.) group.

### Medication and LAAC Procedure

In the procedure for the preparation of LAAC, uninterrupted anticoagulation with either dabigatran or rivaroxaban was performed as typical practice until the operation day. In brief, the occluders were implanted on LAA under the condition of general anesthesia and fluoroscopic guidance *via* the femoral vein and transseptal access. Intraprocedural transesophageal echocardiography (TEE) was performed to identify LAA thrombosis and determine the LAA dimensions for the occluder size. Uninterrupted bolus with unfractionated heparin (UFH) was applied prior to the transseptal puncture with a target activated clotting time (ACT) ≥250 ms. After the procedure, the sheath was removed and hemostasis was achieved with either manual pressure or a figure-of-eight stitch. A routine operation was performed. The patients received successful device implantation. All patients were medicated with a 3-month course of anticoagulation with NOAC for patients to facilitate device endothelialization, followed by dual antiplatelet therapy until 6 months, then lifelong aspirin after discharge. The optimal post-interventional antithrombotic drug regimen as well as treatment duration after LAAC remains a controversial issue (Tilz et al., 2017). The prospective randomized open-label ADRIFT trial initiated the NOAC for 3 months as a comparison to dual antiplatelet therapy, and a decreased thrombin generation was found in rivaroxaban arms (Duthoit et al., 2020). Hence, NOAC has been introduced as a promising novel anticoagulation therapy after LAA occlusion. The schedule for the administration of anticoagulants in our study was based on the ADRIFT study. The anticoagulant treatment was decided by the attending physicians on an individual bleeding risk basis. As for the appearance of DRT formation, the anticoagulant was switched from the NOAC to warfarin within the therapeutic range (INR 2.0–3.0), and additional TEE was scheduled 3 months later.

### Data Collection and Follow-Ups

We collected detailed information about each subject including demographic characteristics, comorbidity disease, laboratory parameters, and concomitant medication through the electronic medical records upon admission.

The scheduled outpatient follow-up visits were performed at 3 months, 6 months, and 12 months after discharge. Repeated TEEs were performed to identify the thrombosis size on occluders, which could reflect the effectiveness of anticoagulant drugs during the follow-up. Routine coagulation function (activated partial thromboplastin time, prothrombin time, fibrinogen, and D-dimer) and blood tests were carried out to assess the bleeding risk by an automatic coagulation analyzer after anticoagulation treatment.

### Clinical Outcomes

We assessed the occurrence of thromboembolic, bleeding events, and thrombus dissolution following adjusted anticoagulant as the primary endpoint between the two groups. The thrombus dissolution by dabigatran or rivaroxaban was represented by the length and width of thrombus formation with the cooperation of initial, secondary, and third TEEs. The thromboembolic events included DRT displayed as an mass with a well-demarcated left atrial boundary of the device defect by TEE- and AF-related systemic embolism at the same time. The bleeding events were classified as major and minor bleeding. Major bleeding was defined as bleeding events causing a reduction in hemoglobin to 20 g/L or more, or leading to transfusion of ≥2 U of blood, or symptomatic bleeding in a critical area, or fatal bleeding. Minor bleeding could be defined as the rest of other bleeding events.

### Statistical Analysis

The descriptive statistics of continuous variables were expressed as means ± standard deviations (SD), and those of discrete variables were expressed as counts or percentages. Student’s t tests were used to compare the differences of the continuous variables among two groups of patients, and chi-squared tests were performed to compare the distribution of categorical variables.

The comparison of thrombosis and bleeding complications were analyzed by Student’s t test. We compared the proportion of patients whose data of coagulation function tests beyond threshold and applied Kaplan–Meier method for survival curves analyses by using the log-rank test for trend and the Cox regression analysis between the two groups. We compared the time to DRT (defined as the time from inclusion to the first occurrence of DRT) between the two groups.

A two-sided *p* value was used to determine the significance (threshold, *p* < 0.05). Statistical analysis was performed using SPSS (IBM SPSS Statistics 22.0) and Prism 5 (GrandPad Software). A *p* value of 0.05 was considered the threshold for statistical significance.

## Results

### Study Population

**FIGURE 1:**
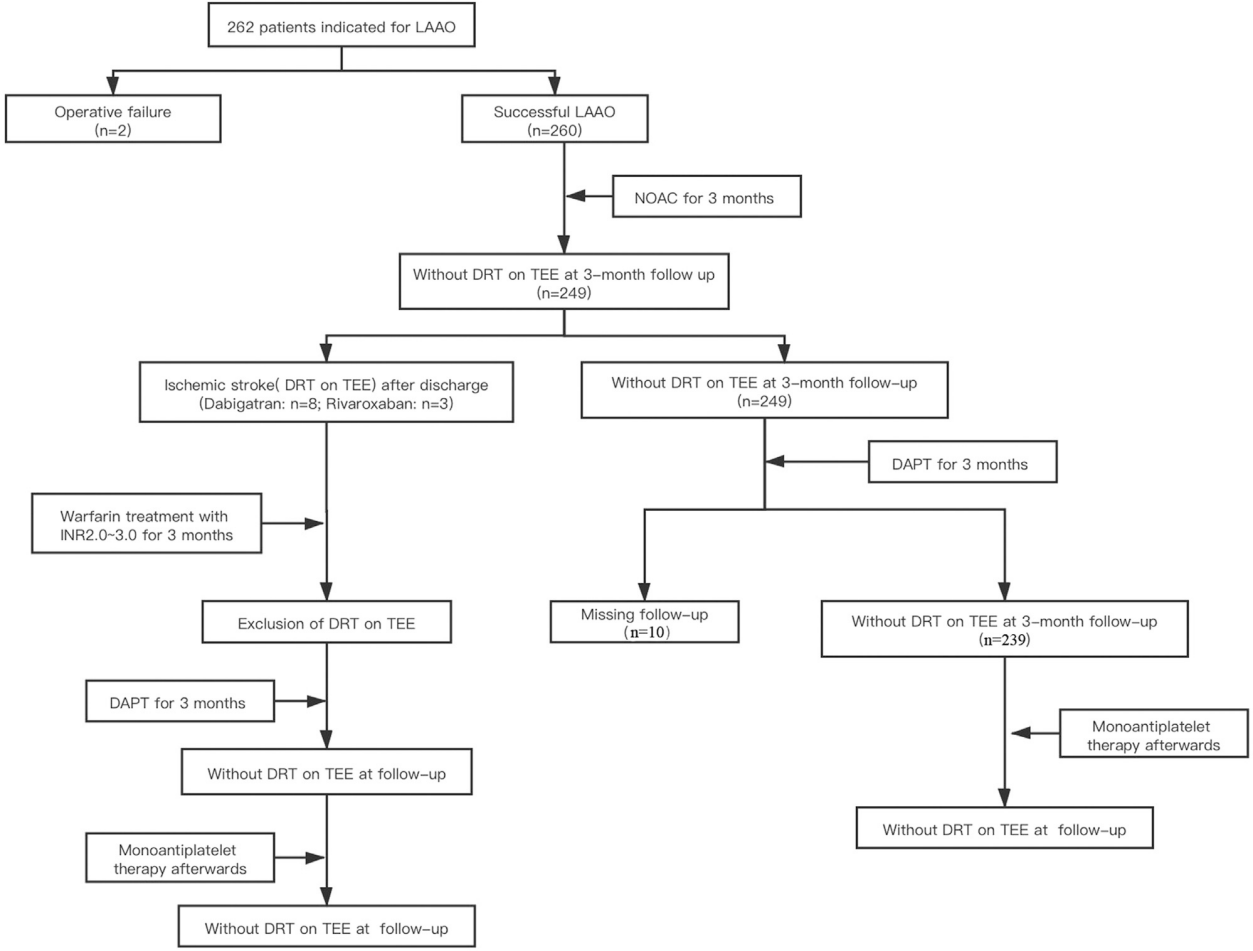
Diagrammatic presentation of our sample size and progression of anticoagulation strategy.

Totally, 262 consecutive NVAF patients who met the inclusion and exclusion criteria were enrolled. Two patients were excluded due to procedure failure, and 260 enrolled patients who underwent LAAC operation successfully completed the 3-month follow-ups with NOAC. During the 3 months post-anticoagulation, a total of 11 DRTs recurred: Eight patients were on dabigatran and three patients were on rivaroxaban at the time of recurrence. The anticoagulants were switched from NOACs to warfarin within the therapeutic range (INR 2.0–3.0), and TEE was performed to identify the resolution of DRT after 3 months following anticoagulation. For patients without DRT on TEE during the 3-month follow-up, they were followed with dual antiplatelet therapy (aspirin plus P_2_Y_12_ receptor antagonist) until 3 months and then lifelong aspirin. Among them, 10 patients were excluded because of the failure of finishing the follow-up. Thus, a total of 250 patients who received NOAC medication after LAAC operation were enrolled in the study. The technical success rate was 95.4%. The research design and progression of anticoagulation therapy for post-LAAC operation are summarized in Figure 1.

NVAF patients who underwent LAAC operation and medicated with NOAC were included in our study. Among the enrolled patients, about 97 received dabigatran (38.8%) and 153 received rivaroxaban (61.2%). The baseline demographic characteristics, comorbidity disease, laboratory parameters, and concomitant medication are summarized in Table 1. No significant difference was found in terms of age, gender, smoking, alcohol, heart rate, hypertension, diabetes mellitus (DM), transient ischemic attack (TIA) or stroke, coronary artery disease, heart failure, coagulation function parameters, and concomitant medication between the two well-matched groups (*p* > 0.05).

**TABLE 1:** Baseline characteristics of the study population.

Baseline characteristics	Dabigatran (N = 97)	Rivaroxaban (N = 153)	*p* Value
Age, years; mean (SD)	69.5 ± 9.1	69.9 ± 8.1	0.704
Gender, male, n (%)	48 (46.6%)	81 (54.3%)	0.186
Smoking, n (%)	13 (12.6%)	13 (8.7%)	0.335
Alcohol, n (%)	9 (8.7%)	17 (11.6%)	0.471
HR (beats·min^−1^)	73.5 ± 8.9	72.5 ± 7.7	0.682
Comorbidities			
Hypertension, n (%)	73 (70.9%)	101 (68.7%)	0.714
Dyslipidemia, n (%)	8 (7.8%)	8 (5.4%)	0.460
Diabetes, n (%)	21 (20.4%)	36 (24.5%)	0.447
CKD, n (%)	25 (24.3%)	30 (20.4%)	0.468
Stroke/TIA, n (%)	45 (43.7%)	74 (50.3%)	0.300
Liver disease, n (%)	12 (11.7%)	19 (12.9%)	0.763
Heart failure, n (%)	3 (2.9%)	2 (1.4%)	0.390
Laboratory tests			
eGFR, mL/(min·1.73m^2^); mean (SD)	71.3 ± 16.4	74.2 ± 17.4	0.182
APTT, s; mean (SD)	31.8 ± 5.8	31.4 ± 6.0	0.591
PT, s; mean (SD)	15.5 ± 6.7	14.6 ± 5.8	0.277
TT, s; mean (SD)	25.8 ± 23.1	22.0 ± 19.3	0.185
D-dimer, mg/L; mean (SD)	0.3 ± 0.3	0.4 ± 0.4	0.307
INR; mean (SD)	1.4 ± 0.6	1.4 ± 0.5	0.485
Co-medication			
Antihypertension, n (%)	56 (54.4%)	72 (49.0%)	0.401
Beta-blocker, n (%)	57 (58.8%)	81 (52.9%)	0.367
PPI, n (%)	10 (9.7%)	24 (16.3%)	0.133
CHA_2_DS_2_-VASc; mean (SD)	3.18 ± 1.6	3.5 ± 1.4	0.160
CHA_2_DS_2_-VASc≥2; n (%)	89 (86.4%)	134 (91.2%)	0.234
CHA_2_DS_2_-VASc≥3; n (%)	65 (63.1%)	105 (71.4%)	0.171
CHA_2_DS_2_-VASc≥4; n (%)	40 (38.8%)	75 (51.0%)	0.057
HAS-BLED; mean (SD)	3.0 ± 1.5	3.2 ± 1.3	0.401
HAS-BLED≥3; n (%)	67 (65.0%)	110 (74.8%)	0.094

The data are shown as mean (SD) or %. APTT: activated partial thromboplastin time, CKD: chronic kidney disease, eGFR: estimated glomerular filtration rate, HR: heart rate, INR: international normalized ratio, PT: prothrombin time, SD: standard deviation, TIA: transient ischemic attack, TT: thrombin time; antihypertension was referred as ACEI and ARB; PPI: proton-pump inhibitor; thrombosis and bleeding risk was represented with CHA_2_DS_2_-VASc and HAS-BLED score, respectively.

The ratio with high thromboembolic risk according to a CHA_2_DS_2_-VASc score of >4 was 38.8 and 51.0% for dabigatran and rivaroxaban, respectively. The bleeding risk with a HAS-BLED score of >3 was 65.0 and 74.8% of cases for dabigatran and rivaroxaban anticoagulation.

### LAAC Procedure Characteristics

Imaging analysis during the LAAC operation procedure identified a mean diameter of the LAA with (24.6 ± 3.6 mm). The mean size of the occluders was 29.8 ± 3.6 mm. A comparison of main operation procedural data is shown in Table 2.

**TABLE 2:** Details of LAAC operation characteristics between rivaroxaban and dabigatran groups.

LAAC operation characteristics	Dabigatran (N = 97)	Rivaroxaban (N = 153)	*p* Value
Procedures course			
Duration, min	66.5 ± 17.8	67.1 ± 18.1	0.792
UFH, U	5456.2 ± 754.2	5279.6 ± 759.1	0.074
Diameter of LAA, mm	24.6 ± 3.5	24.6 ± 3.6	0.938
Device diameter, mm	29.6 ± 3.5	29.8 ± 3.7	0.661

Duration time was shown as the whole LAAC operation; UFH: unfractionated heparin.

### Timing of DRT After LAAC During Follow-Ups

**FIGURE 2:**
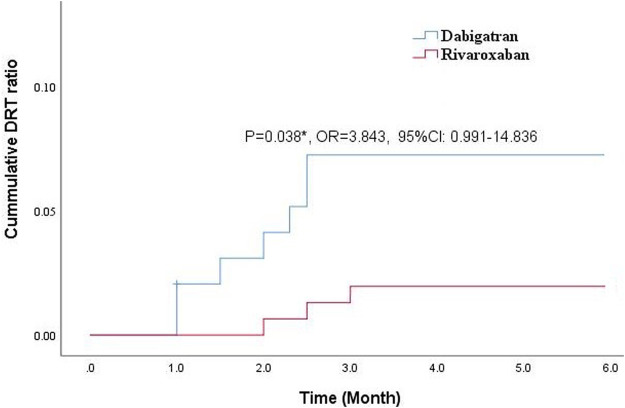
Comparison of cumulative DRT ratio between dabigatran and rivaroxaban during the 3-month follow-up.

Clinical and TEE imaging follow-ups were available for all enrolled patients at 3 months. During the follow-up period, three patients (1.9%) in the rivaroxaban group and eight (8.2%) in the dabigatran group were experiencing DRT events. Cumulative Kaplan–Meier estimates illustrated that the incidence of the DRT was lower for rivaroxaban treatment and reached a significant difference during the 6-month follow-ups (*p* = 0.038*, OR = 3.843, 95% CI: 0.991–14.836). In the whole cohort of rivaroxaban-treated patients, those with dabigatran medication were significantly more likely to experience shorter time to DRT, as shown in Figure 2.

### Clinical Outcomes Assessments

During the 3-month anticoagulation follow-up, we observed no substantial differences between the two groups in terms of incidence of systemic thromboembolism including stroke and cardiac embolism for LAAC patients (overall *p* = 0.468, with composite endpoint rates of 9.2 and 6.5% for dabigatran and rivaroxaban treatment, respectively). No significant difference was observed between the two groups when it comes to the occurrence ratio of left atrial dilation defined as enlargement of the left atrial diameter >40 mm and LVEF <40%. Also, there was no significant difference between the two groups with respect to the levels of coagulation parameters as PT and APTT (Table 3).

**TABLE 3:** Clinical outcomes and coagulation parameters comparison.

	Dabigatran (N = 97)	Rivaroxaban (N = 153)	*p* (%)	OR	95%CI
Systemic embolism, (%)	9.2%	6.5	0.468	0.474	0.464–0.483
Stroke, (%)	5.1%	3.2	0.462	1.609	0.453–5.709
Cardiac embolism, (%)	4.1%	3.3	0.724	1.273	0.333–4.863
TEE on follow-up					
LVEF (<40%, %)	88.6%	81.0	0.244	1.506	0.756–3.003
LA (>40mm, %)	0	1.3	0.646	0.629	0.087–4.542
Coagulation parameters					
APTT >31s, (%)	60.8%	46.4	0.566	1.161	0.697–1.935
PT > 13s, (%)	48.4%	44.4	0.045	1.697	1.013–2.842

APTT: activated partial thromboplastin time, LA: left atrial, LVEF: left ventricular ejection fraction, PT: prothrombin time; systemic embolism is defined as stroke and cardiac embolism.

### The Resolution of Thrombus Formation on Closure

**FIGURE 3:**
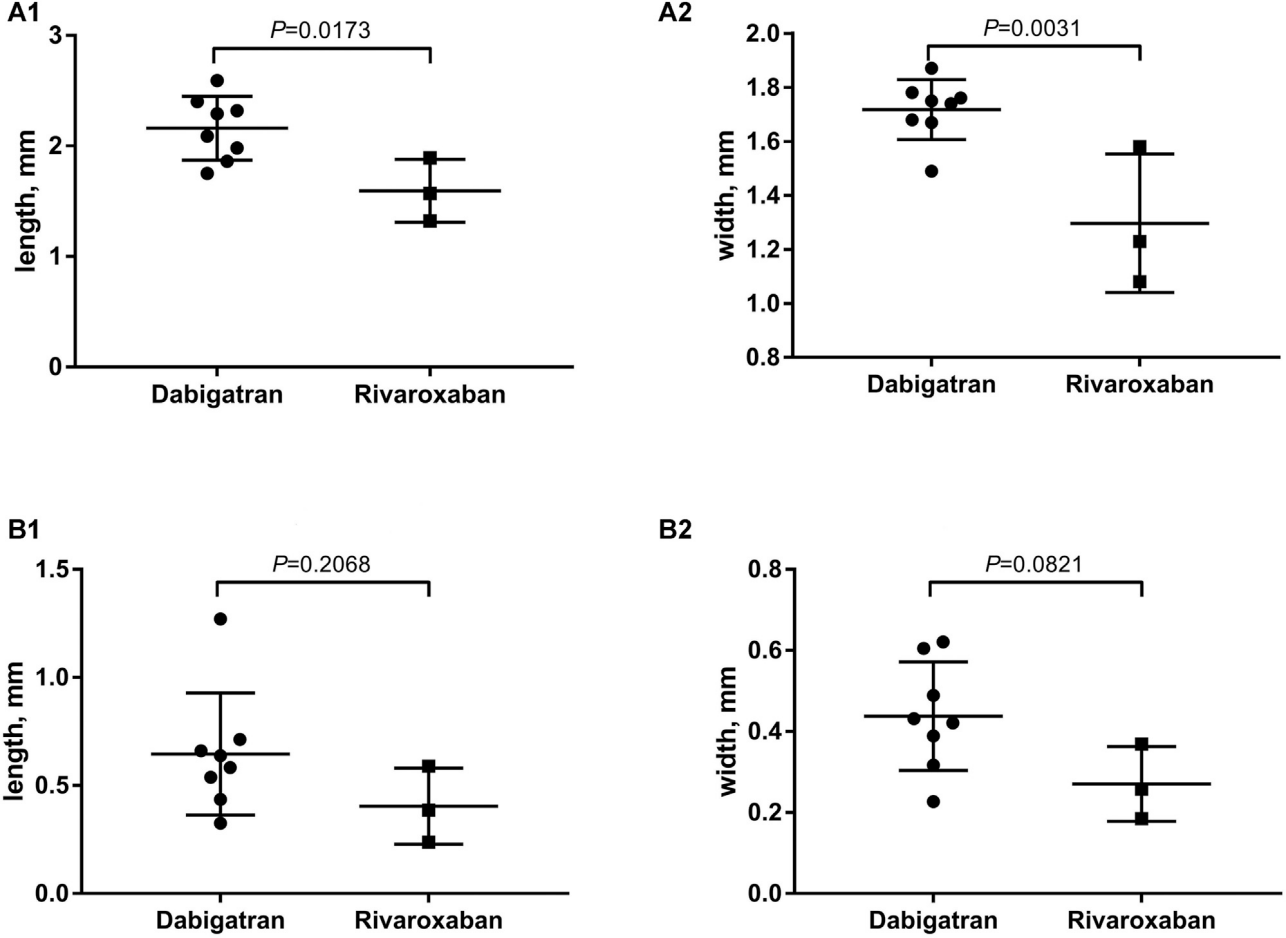
**(A)** The average of length and width for DRT formation with the cooperation of initial TEE after the 3-month follow-up; **(B)** elimination of thrombosis after the switch from dabigatran or rivaroxaban to warfarin.

The thrombus formation size on the device was represented by length and width with the cooperation of initial TEE after the 3-month follow-up. The TEE results showed that the average length and width of DRT in the rivaroxaban group was significantly lower compared with that in the dabigatran group (2.16 vs. 1.60 mm, *p* = 0.017*, and 1.71 vs. 1.30 mm, *p* = 0.003*, respectively) (Figure 3A).

After the switch from dabigatran or rivaroxaban to warfarin within the therapeutic range (INR 2.0–3.0), the scheduled 3-month follow-up TEE showed an elimination of thrombosis (Figure 3B). The comparison of thrombosis formation and dissolution for dabigatran or rivaroxaban was represented by the average length and width of thrombus with the cooperation of secondary TEE (0.64 vs. 0.40 mm, *p* = 0.206, and 0.43 vs. 0.27 mm, *p* = 0.082, respectively).

### Anticoagulation-Related Complications

The frequencies of bleeding events in the dabigatran and rivaroxaban groups are shown in Table 4. Overall, the most common bleeding events were prone to operation site hemorrhage during anticoagulation with dabigatran than those in the rivaroxaban group (2.1 vs. 1.3%), with no significant difference among the two groups (*p* = 0.646). The cumulative incidences of bleeding complications such as gastrointestinal hemorrhage and skin ecchymosis during anticoagulation therapy were also similar in the two groups (*p* > 0.05). There was no significant difference between the two groups with respect to the levels of Hb and PLT under the bleeding threshold (*p* > 0.05). There was no significant difference in the time of hospitalization during anticoagulation with rivaroxaban when compared with dabigatran (*p* = 0.432, HR: 0.432, 95% CI: 0.374–0.948), as shown in Table 4.

**TABLE 4:** Anticoagulation complications comparison with NOACs for LAAC.

	Dabigatran (N = 97)	Rivaroxaban (N = 153)	*p* Value	OR	95%CI
Bleeding complications					
Gastrointestinal hemorrhage, (%)	5.1%	5.2%	0.979	0.985	0.313–3.103
Operation site hemorrhage, (%)	2.1%	1.3%	0.646	1.589	0.220–11.474
Skin ecchymosis, (%)	1.1%	1.3%	0.845	0.786	0.070–8.792
Laboratory parameters					
PLT <125, (%)	8.2%	7.2%	0.759	1.160	0.449–2.996
Male: Hb < 120, (%)	4.1%	4.0%	0.937	1.054	0.290–3.834
Female: Hb < 110, (%)					
Days of hospitalization, (days)	5.9 ± 2.1	5.6 ± 3.1	0.432	0.856	0.374–0.948

PLT: platelet count, Hb: hemoglobin.

## Discussion

To the best of our knowledge, this is the first study to investigate clinical efficacy associated with dabigatran and rivaroxaban exposure for patients who underwent percutaneous LAA occluder implantation. The main findings of the present study are the following: (1) postoperative DRT occurrence was higher during the anticoagulation course of dabigatran than rivaroxaban and (2) it is effective to transfer to warfarin as a resolution therapy on the DRT.

Previous literature had provided definitive evidence on the clinical benefit of rivaroxaban for post-anticoagulation of LAAC (Enomoto et al., 2017; Duthoit et al., 2020). However, due to the lack of comparative studies on different NOACs (mainly dabigatran and rivaroxaban), the optimal anticoagulant remains uncertain. According to the PROTECT AF clinical trial, the DRT occurrence ratio was reported to be observed about 4.2% for successfully occluder implanted patients (Reddy et al., 2011). In our population, the total DRT ratio remained similar to the previous study with about 4.4% among LAAC operation patients. However, the overall DRT incidence was found to be 1.9% (3/153) under anticoagulation with rivaroxaban occurring less frequently as compared to dabigatran anticoagulation with DRT ratio of 8.2%, suggesting that dabigatran is less effective than rivaroxaban in reducing thrombosis after LAAC procedures. One probable explanation might be that common genetic variants of CES1 and ABCB1 have been identified to potentially account for the interindividual variations in dabigatran plasma levels, which could lead to varied anticoagulation therapeutic responses (Dimatteo et al., 2016; Gouin-Thibault et al., 2017). It has been proven that the single nucleotide polymorphism (SNP) in the CES1 gene (rs2244613) could alter dabigatran metabolism, leading to lower trough concentrations and increasing thrombosis risks (Merali et al., 2014; Nakamura et al., 2019). Contrary to the anticoagulant mechanism of dabigatran, rivaroxaban is a factor Xa inhibitor that can selectively inhibit FXa and has a rapid onset of action, which could help prevent thrombosis and platelet aggregation (Anand et al., 2018; Petzold et al., 2020). Many clinical trials had shown that administration of rivaroxaban combined with antiplatelet therapy could reduce the incidence of thromboembolic events, including cardiovascular events, myocardial infarction, and stroke due to the reduction of thrombosis risks (Eikelboom et al., 2017; Korjian et al., 2019).

Our results showed that the DRT incidence was more uncommon under anticoagulation with rivaroxaban at 3 months (3 patients) as compared to dabigatran (8 patients), which indicated that early rivaroxaban anticoagulation might be more protective for LAAC operation (*p* = 0.038*). Meanwhile, the marked increasing incidence of DRT at 1, 2, and 3 months for post-anticoagulation with dabigatran after LAA occluder implantation suggested that dabigatran might decrease endothelialization in some LAAC operation cases. Currently, no relevant literature was reported on the increasing risks of thrombosis after LAAC operation under dabigatran anticoagulation. One previous clinical study demonstrated that dabigatran could increase myocardial infarction risks due to increasing platelet activity *via* enhancing the thrombin receptor density on thrombocytes (Achilles et al., 2017). The enhanced platelet reactivity of dabigatran induced by thrombin receptor-activating peptide is a characteristic of the thrombin-induced platelet activation (Olivier et al., 2016; Vinholt et al., 2017). This might be one of the reasons for increasing occurrence of DRT after LAAC operation under dabigatran anticoagulation.

In the present study, we investigated the thrombosis size that might reflect an increased subsequent risk of thromboembolic events under different NOACs. The average length and width of thrombus was significantly lower in the rivaroxaban group compared to the dabigatran group after the 3-month follow-up. One previous study indicated that rivaroxaban could rapidly decrease coagulation parameters after tablet intake, and contribute to lower levels of prothrombin fragments as compared to dabigatran (Duthoit et al., 2020). The recent clinical studies indicated that the Xa inhibitor, rivaroxaban, might be a potential anticoagulant for the resolution of DRT for LAAC operation (Enomoto et al., 2017). Also, a previous study showed the superiority of rivaroxaban on the resolution of LAA thrombus in NVAF patients compared with warfarin (Ke et al., 2019).

Besides the antithrombosis effect, the safety profile such as bleeding complications of two different NOACs needs to be taken into account. Our results showed that no significant difference was found between groups with respect to laboratory biomarkers such as Hb, Hct, and PLT (*p* > 0.05). Our findings are largely consistent with a direct comparison study that tended to demonstrate similar safety between dabigatran and rivaroxaban (Noseworthy et al., 2016).

The other important observation in this study was that the rate of cardiac dysfunction was comparable to that of the patients who received NOACs, both peri-procedurally and early during the follow-up. We were not able to detect a discernible benefit of anticoagulation with rivaroxaban over dabigatran regarding clinical endpoints including systemic embolism such as stroke and cardiac embolism in patients receiving LAAC operation. One research indicated that no differences were found between the two NOACs in the risk of stroke or systemic embolism (Noseworthy et al., 2016). Left atrial size enlargement and LVEF were predictors of mortality for both cardiovascular issues and all-cause mortality. Our findings suggested that rivaroxaban showed no priority over dabigatran in terms of cardiac function parameters. The main explanation might be that the combination medication with renin–angiotensin–aldosterone system (RAAS) inhibitors and beta-blockers appeared to prevent the new-onset atrial fibrillation in patients with left ventricular dysfunction.

## Conclusion

In summary, our results provide a significant addition to the previous literature, since the existing studies are mainly limited to comparisons of efficacy and safety between NOACs for post-LAAC anticoagulation. We demonstrate that rivaroxaban administration after LAAC operations might be a more advantageous alternative, which could prevent DRT complications without increasing the risk of hemorrhage as compared to dabigatran.

## Limitations

First, it was relatively difficult to make an accurate conclusion as for a single and observational study. Large prospective and randomized controlled trials are required to assess clinical outcomes in the future. Second, the study might be impractical due to the very low incidence of DRT. Hence, a large sample size is needed in a further study. Third, we did not assess the potential clinical significance of DRT in the occluder implantation population mainly due to the lower incidence of DRT and thromboembolic events. Finally, the DRT occurrence was only collected at the first time of follow-up TEE detection postoperatively. Many case reports have confirmed that DRT can be found early after implantation, which might affect the observation time of DRT.

## Data Availability

The datasets presented in this study can be found in online repositories. The names of the repository/repositories and accession number(s) can be found in the article/Supplementary Material.
